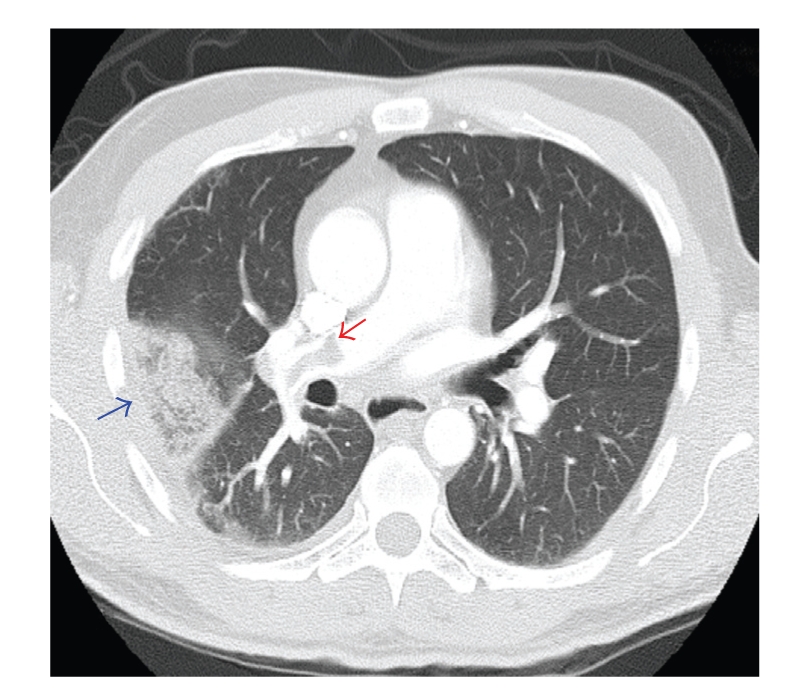# Pulmonary Embolism Mimicking Pneumonia in a HIV Patient

**DOI:** 10.1155/2010/394546

**Published:** 2010-06-14

**Authors:** Vivek Nagaraja, Joel A. Terriquez, Hemanth Gavini, Lokesh Jha, Stephen A. Klotz

**Affiliations:** ^1^Department of Internal Medicine, University Physicians Healthcare, Tucson, AZ, USA; ^2^Section of Infectious Diseases, Department of Medicine, University of Arizona, Tucson, AZ, USA

## Abstract

Recent studies have shown an increased risk of arterial and venous vascular diseases in HIV patients, pulmonary thromboembolism being one of them. HIV-infected individuals may have procoagulants predisposing them to thromboembolism. Patients with thromboembolism may have a clinical presentation mimicking common opportunistic infections. It is important to consider pulmonary embolism in the differential of HIV patients with fever, cough, and dyspnea, particularly in those with well-controlled HIV infection.

## 1. Introduction

Infection with the human immunodeficiency virus (HIV) is now a chronic disease in the developed world. HIV-infected patients have a better life expectancy with the advent of highly potent antiretroviral therapy (ARV) than in the past. However, chronic HIV infection and ARVs are now associated with long-term complications [1]. In recent literature, the relationship between HIV infection and cardiovascular disease has been addressed. Studies have shown an increased risk of arterial and venous vascular diseases in HIV patients. For example, a cohort study comparing 4993 HIV patients revealed an incidence of myocardial infarction of 0.59 to 3.41/1000 patient-years [2], another with 23,468 HIV-positive patients found an incidence of myocardial infarction of 3.5/1000 patient-years [3]. In a systematic review of literature from 1986 to 2004, Klein et al. reported that the incidence of venous thrombotic events in HIV-infected patients was increased two to ten-fold in comparison with healthy individuals of similar age [1]. In a series of HIV-positive patients with venous or arterial thrombosis, pulmonary emboli accounted for 66% of all thrombotic events [4].

## 2. Case Presentation

A 41-year-old man with a history of HIV (diagnosed in 1988) on ARVs presented with three days of non-productive cough, intermittent fever, chills, dyspnea on exertion, and generalized fatigue. His past medical history included *Cytomegalovirus* retinitis with bilateral blindness, Kaposi's sarcoma, lipodystrophy and lipoatrophy, hypothyroidism, and hypertension. His medications included abacavir/lamivudine, ritonavir, atazanavir, pravastatin, metformin, levothyroxine, and valsartan. The patient's last CD4 cell count was 800 cells/mm^3^, and the viral load was undetectable five months previously while on his current antiretroviral regimen.

 The patient was febrile at 100.8°F, pulse of 118/min, blood pressure 93/65 mm of Hg, respiratory rate 18/min, and oxygen saturation of 93% on room air. The chest was clear with normal heart sounds on auscultation. He was treated with intravenous ceftriaxone and trimethoprim/sulfamethoxazole for presumed community-acquired pneumonia and possible PCP. The complete blood count and comprehensive metabolic panel were unremarkable. The chest radiograph revealed normal lung fields. Blood cultures were negative. A high resolution CT scan of the chest was performed and was normal. The patient reported subjective clinical improvement after 48 hours of hospitalization. He was discharged with a diagnosis of a probable viral upper respiratory tract infection.

 One week later the patient returned to the emergency department with three days of intermittent fever, cough, and dyspnea. The CD4 cell count obtained during the previous admission returned at 169 cells/mm^3^, with a nondetectable viral load. His blood pressure was 97/62 mm of Hg, temperature 99.9°F, heart rate 131 beats/minute and respiratory rate of 20 breaths/minute, with an oxygen saturation of 96% on room air and 95% after walking. On physical examination the patient was dehydrated. The rest of the examination was unremarkable. 

 The complete blood count, comprehensive metabolic profile, and the chest radiograph were normal. A repeat CT scan of the chest without contrast revealed a peripherally located patchy airspace opacification on the right side. Blood cultures showed no growth. Bronchoalveolar lavage was performed and cultures were negative for bacterial, viral, mycobacterial, or fungal growth. A Doppler ultrasound of both lower extremities did not show evidence of DVT. A CT scan of the chest with contrast (Figure 1) was performed to evaluate for pulmonary embolism due to the persistent cough. It revealed filling defects consistent with pulmonary embolism in the right main pulmonary artery [red arrow] and large arterial branches to the right upper, middle, and lower lobes and smaller emboli in the distal branches of the left lower lobe as well. The peripheral opacity in the right lung field was a wedge infarct [blue arrow]. 

 Antibiotics were discontinued and the patient was started on intravenous heparin and coumadin. The patient is doing well 6 months after hospitalization.

## 3. Discussion

The synergistic interplay of multiple risk factors may contribute to the increased risk of developing a thrombo-embolic phenomenon in patients with chronic HIV infection. These include coagulation abnormalities, presence of opportunistic infections, malignancies [5], and severity of HIV infection.

 A number of abnormalities of coagulation have been described in patients with HIV infection. The most common is the lupus anticoagulant found in up to 60% of the patients and may be associated with major thrombo-embolic phenomena [6]. The increase in procoagulant factors like tissue factor and elevated concentrations of microparticles are important contributing factors. Microparticles are relatively small cellular remnants circulating in plasma originating from platelets and endothelial cells. In HIV patients, microparticles also originate from CD4+ lymphocytes, as a direct consequence of HIV infection and possibly as a reflection of CD4+ lymphocyte apoptosis [1]. Other coagulation abnormalities include reduced levels of antithrombin III, activated protein S [1, 7], and activated protein C [8], acquired heparin cofactor II deficiency [9] and increased levels of fibrinogen, d-dimer, Von-Willibrand factor, plasminogen activator inhibitor-1, and tissue-type plasminogen activator antigen [10].

 Two epidemiological studies showed higher risk of venous thromboembolism in patients with CD4 cell count less than 200 cells/mm^3^. This level of CD4 count predisposes to many opportunistic infections further contributing to a procoagulant state through enhancing the active ongoing immune response. Several opportunistic infections, particularly *Cytomegalovirus* and *Herpes simplex* virus types 1 and 2, may contribute to the prothrombotic state by converting vascular endothelial cells from a noncoagulative to a procoagulative phenotype, leading to expression of procoagulant phospholipids [8]. An association between cigarette smoking and spontaneous thrombosis in HIV patients has been mentioned [11] and was reported to affect 77% of the patients [12]. 

Pulmonary embolism can masquerade pneumonia. In patients with a new diagnosis of pulmonary embolism but with no previous history of thromboembolic or cardiopulmonary disorders, cough (28%), pleuritic pain (57%), fever (3%), and abnormal lung examination (26%) [13] may be present at initial presentation mimicking a pneumonia or opportunistic pulmonary infection. 

 The use of protease inhibitors has also been implicated in hypercoaguability [1, 8, 14]. Majluf-Cruz et al. [8] found that the incidence of venous thrombotic events increased dramatically from 0.19% before the introduction of protease inhibitors to 1.07% afterwards. An interesting suggestion by Saif et al. [15] was that because aspartyl proteases such as renin, endothelin, and cathepsin D are involved in the regulation of coagulation, and because the HIV protease is an aspartyl protease, inhibition of these native proteases by HIV-protease inhibitors may lead to a prothrombotic state. 

 The relationship between HIV infection and the development of thrombotic events, either arterial or venous, is dependent on the interplay amongst multiple factors, which include coagulation abnormalities, status of immunity, and ARVs. Because, available evidence supporting the above relationship is limited, more research is required in this area. Physicians need to be aware of the increased risk of thrombo-embolic disorders in HIV-infected patients so that they can be appropriately recognized and treated.

## Figures and Tables

**Figure 1:**